# Integrative diagnosis of invasive pulmonary aspergillosis in non-neutropenic patients using BALF-tNGS–derived *Aspergillus* load and host risk factors: a multicenter study

**DOI:** 10.3389/fcimb.2026.1739837

**Published:** 2026-02-12

**Authors:** Furui Liu, Bofei Liu, Jinyan Wang, Zhifeng Wang, Wenyan Zhou, Yonghong Yang, Wenling Chen, Ying Yang, Tao Feng, Jinyuan Zhu

**Affiliations:** 1 The First School of Clinical Medicine, Ningxia Medical University, Yinchuan, Ningxia, China; 2 The Fourth People’s Hospital of Ningxia Hui Autonomous Region, Yinchuan, Ningxia, China; 3 The Third People’s Hospital of Ningxia, Yinchuan, Ningxia, China; 4 Department of Critical Care Medicine, General Hospital of Ningxia Medical University, Yinchuan, China

**Keywords:** bronchoalveolar lavage fluid, invasive pulmonary aspergillosis, multicenter study, non-neutropenic, targeted next-generation sequencing

## Abstract

**Background:**

Diagnosing invasive pulmonary aspergillosis (IPA) in non-neutropenic patients is challenging because of non-specific manifestations and limited diagnostic tools. Targeted next-generation sequencing (tNGS) of bronchoalveolar lavage fluid (BALF) enables rapid pathogen detection; however, its capacity for quantitative assessment of fungal load remains unclear. This study integrated BALF-tNGS fungal load with host risk factors to develop a diagnostic nomogram for IPA in non-neutropenic patients.

**Methods:**

Non-neutropenic adults with suspected IPA were retrospectively enrolled at three tertiary hospitals (December 2020–December 2024). IPA was classified according to consensus definitions. Normalized Aspergillus reads from BALF-tNGS were stratified into low, medium, and high tiers. Clinical, radiological, and microbiological variables were analyzed, and a multivariable logistic regression model was built and internally validated via bootstrapping. Diagnostic performance was assessed using receiver operating characteristic analysis.

**Results:**

Among 238 patients, 134 had IPA and 104 had non-IPA. Compared with non-IPA cases, patients with IPA had higher rates of diabetes (73.9%), corticosteroid exposure (87.3%), bacterial co-infection (94.8%), and intensive care unit (ICU) admission (72.4%) (all *P* < 0.01). IPA prevalence peaked in the medium-load group (75.4%) compared with the low-load (36.7%) and high-load (38.3%) groups (*P* < 0.001). Seven independent predictors, *Aspergillus* load, diabetes, corticosteroid exposure, bacterial co-infection, ICU admission, nodular shadow, and positive BALF culture, were incorporated into the model, which showed excellent discrimination (AUC = 0.966; sensitivity, 91.8%; specificity, 95.2%) and good calibration.

**Conclusion:**

Integrating BALF-tNGS–derived normalized *Aspergillus* reads with host factors substantially improves differentiation of IPA from colonization in non-neutropenic patients. This semi-quantitative framework supports early, individualized antifungal decision-making and merits external validation.

## Introduction

*Aspergillus* conidia are ubiquitous airborne spores that are continuously released into the environment. These spores can readily penetrate the alveoli, making the lungs the primary site of infection and leading to invasive pulmonary aspergillosis (IPA) (Bulpa et al., 2007). Historically, IPA was regarded as a disease largely confined to individuals with neutropenia or other forms of immunocompromise. However, recent epidemiological studies have shown a steady increase in incidence among non-neutropenic patients (Latgé and Chamilos, 2019). Early diagnosis of IPA remains particularly challenging. In non-neutropenic populations, the absence of specific clinical manifestations, coupled with the limited sensitivity and specificity of conventional microbiological assays, such as the serum galactomannan test, frequently results in delayed diagnosis and suboptimal treatment (Jenks et al., 2019).

Conventional diagnostic approaches for *Aspergillus* detection include fungal culture and direct microscopic examination of bronchoalveolar lavage fluid (BALF) or sputum, as well as histopathological evaluation of lung biopsy specimens (Ascioglu et al., 2002). However, these methods have notable limitations. Culture, although considered a diagnostic reference standard, is time-consuming, requires viable organisms, and has a low positive yield because of the fastidious growth of *Aspergillus* spp. and the influence of prior antifungal therapy. Moreover, the turnaround time for histopathology is long, and invasive sampling carries substantial procedural risk. Although direct microscopy of BALF or sputum provides a rapid and simple alternative, its diagnostic performance is suboptimal, with substantial variability in both sensitivity and specificity (Denning et al., 2011). In addition, metagenomic next-generation sequencing (mNGS) enables the unbiased detection of diverse pathogens, including rare ones (Miller et al., 2019; Ramachandran and Wilson, 2020). However, its clinical adoption remains limited by high costs, host DNA interference, dual DNA/RNA workflows, and the absence of standardized protocols or large-scale validation, leading to subjective interpretation of results (Zheng et al., 2021).

Recently, highly multiplexed polymerase chain reaction–based targeted next-generation sequencing (tNGS) has emerged in China as a promising complement or alternative to mNGS for infectious disease diagnostics. This approach utilizes curated panels that encompass clinically relevant pathogens, thereby enhancing analytical sensitivity and enabling detection even in specimens with low microbial loads (Mamanova et al., 2010; Dennis et al., 2022). Targeted amplification also mitigates interference from host background sequences, improving data efficiency and reducing costs. However, quantitative sequencing reads generated by tNGS should not be interpreted as independent markers of pathogenicity; rather, they must be integrated with clinical findings and ancillary diagnostic evidence (Gu et al., 2019). Interpretation based solely on absolute read counts remains controversial due to substantial inter-platform variability (Lee et al., 2014; Khamina et al., 2022). Notably, tNGS (Deeplex) performance in cerebrospinal fluid has been shown to correlate strongly with bacterial load; the probability of Mycobacterium tuberculosis detection decreased markedly with increasing Xpert cycle threshold (Ct) values, with stepwise differences in read depth across Ct strata (Tram et al., 2024). This finding indicates that tNGS read depth and Xpert Ct have comparable quantitative and clinical significance. Accordingly, integrating normalized *Aspergillus* read counts from BALF-tNGS, stratified into cohort-based low, medium, and high tiers, into diagnostic models may improve discrimination of IPA from airway colonization in non-neutropenic patients.

Therefore, this multicenter retrospective study aimed to develop and validate an integrated diagnostic model that combines BALF-tNGS data with key host clinical factors to differentiate IPA from airway colonization in non-neutropenic patients. By incorporating normalized *Aspergillus* read counts as a semi-quantitative indicator of fungal burden, the study sought to enhance diagnostic accuracy, improve clinical decision-making, and provide a practical framework for interpreting tNGS results in real-world settings.

## Methods

### Subjects

Ethical approval was obtained, with a waiver of informed consent due to the study’s retrospective design. Between December 2020 and December 2024, non-neutropenic patients with pulmonary infections admitted to three tertiary care hospitals in China were consecutively enrolled in this multicenter, retrospective cohort study.

Eligible participants were 18 years of age or older and met the diagnostic criteria for IPA as defined by established international guidelines (Bassetti et al., 2024). Confirmed IPA was defined by either histopathologic evidence of hyphal invasion from a sterile site or a positive culture for *Aspergillus* spp. from a sterile specimen. Probable IPA required all three of the following components: (1) host factors, including underlying conditions such as post-influenza or post–COVID-19 infection, chronic obstructive pulmonary disease (COPD), or hematologic malignancy; (2) clinical and radiologic criteria, consisting of compatible manifestations such as refractory fever or progressive respiratory decline in conjunction with imaging findings indicative of pulmonary infiltrates or cavitary lesions; and (3) mycologic criteria, defined as microbiologic evidence of infection, including a positive *Aspergillus* culture from BALF or a galactomannan (GM) optical density index > 0.5 in serum or ≥ 1.0 in BALF.

Patients with profound immunosuppression (e.g., those who have undergone solid-organ or hematopoietic stem-cell transplantation) were excluded.

Participants were classified into an IPA group (proven or probable IPA) or a non-IPA group (Bassetti et al., 2024) (**Supplementary Table 1**). The non-IPA group included patients with alternative final diagnoses, such as bacterial pneumonia or organizing pneumonia, as well as patients with *Aspergillus* airway colonization in the absence of evidence of invasive disease.

BALF galactomannan was measured using the Platelia™ *Aspergillus* Ag assay (Bio-Rad), and serum or BALF 1,3-β-D-glucan was determined using the Fungitell^®^ assay (Associates of Cape Cod). Bronchoalveolar lavage procedures were performed with a standardized instillation volume of 150–200 mL of saline, with a target fluid recovery rate of >40%.

### Data collection

Clinical and pathological data were systematically collected from all participating centers, including patient demographics, comorbidities, arterial blood gas analyses (assessed by the arterial oxygen partial pressure/fraction of inspired oxygen [PaO_2_/FiO_2_]), and laboratory findings, such as complete blood count, C-reactive protein, and procalcitonin levels. Microbiological results were recorded in parallel. Chest computed tomography images were independently evaluated and reported by experienced, board-certified radiologists.

### tNGS analysis and *Aspergillus* quantification

tNGS was performed by a centralized reference laboratory (KingMed Diagnostics, Guangzhou, China) using the RP100™ Respiratory Pathogen Multiplex Testing Kit (KingCreate Bio, Guangzhou, China) (Yin et al., 2024). This multiplex PCR–based platform integrates broad-spectrum amplification with high-throughput sequencing to enable the simultaneous detection of up to 198 respiratory pathogens, including bacteria, viruses, and fungi.

Briefly, total nucleic acids were extracted from BALF specimens and quantified to ensure standardized input prior to library preparation. Target-specific multiplex PCR amplification was then conducted using curated primer sets covering conserved and species-informative genomic regions. Libraries that met predefined quality-control thresholds were subjected to single-end sequencing on an Illumina platform, with low sequencing depth per sample, consistent with the targeted design of the assay.

According to manufacturer validation and independent analytical evaluations, the limit of detection of the RP100 panel for *Aspergillus* spp. is ≤50 genomic copies per reaction for major pathogenic species, including *Aspergillus fumigatus* and *Aspergillus flavus*, as determined using standardized reference materials (Yin et al., 2024). The fungal targets include both pan-*Aspergillus* loci and species-specific markers, allowing sensitive detection and broad differentiation within the genus. Each sequencing run incorporated negative controls (nuclease-free water) to monitor contamination and positive controls consisting of synthetic pathogen DNA to verify assay performance.

Raw sequencing reads underwent quality filtering to remove low-quality and adaptor-contaminated reads, after which high-quality reads were aligned to a curated microbial reference database. To account for inter-sample variability in sequencing depth, pathogen abundance was normalized as reads per million (RPM) high-quality reads. For each BALF specimen, normalized *Aspergillus* abundance was calculated by dividing the number of reads uniquely mapped to *Aspergillus* spp. by the total number of high-quality reads and multiplying by 1,000,000.

Unlike quantitative PCR–based assays, tNGS does not generate cycle threshold values. Instead, sequencing read counts reflect a composite signal influenced by the initial nucleic-acid burden and the efficiency of multiplex PCR amplification. Consequently, normalized read depth was interpreted as a semi-quantitative indicator rather than an absolute measure of fungal burden.

To facilitate comparative and regression analyses, patients were stratified into three categories based on the cohort distribution of normalized *Aspergillus* RPM values: low load (<177 RPM), medium load (177–6,025 RPM), and high load (>6,025 RPM), corresponding to the 25th and 75th percentiles of the study population. This percentile-based stratification approach was adopted to minimize platform-specific bias and to enable clinically interpretable integration of molecular fungal signals with host risk factors.

### Statistical analysis

All analyses were conducted using SPSS v27.0 and R v4.5.1. Categorical variables were summarized as n (%) and continuous variables as mean ± standard deviation (SD) or median (interquartile range [IQR]), as appropriate. Normality was assessed using the Shapiro–Wilk test. Between-group comparisons employed Student’s t test, ANOVA, or Kruskal–Wallis test for continuous variables, depending on distribution and variance homogeneity, and χ² or Fisher’s exact tests for categorical variables. The multivariable model included seven predictors for 134 IPA events, the less frequent outcome, corresponding to an events-per-variable ratio of approximately 19:1, exceeding the commonly accepted conservative threshold of 10:1. Analyses were performed using complete cases. Missing data for variables included in the model were minimal (<2%) and were handled by listwise deletion.

Variables with *P* < 0.1 in univariate analyses were entered into multivariate logistic regression using a stepwise selection approach. In regression analyses, *Aspergillus* level was modeled as an ordinal categorical variable with three tiers derived from percentile-based stratification of normalized BALF-tNGS RPM values. Internal validation was conducted via bootstrapping: the complete modeling procedure, including variable selection and coefficient estimation, was repeated on 1,000 bootstrap samples drawn with replacement. Model performance metrics, such as area under the receiver-operating characteristic curve (AUC), sensitivity, specificity, positive predictive value (PPV), and negative predictive value (NPV), were computed for each bootstrap iteration and averaged to obtain optimism-corrected estimates.

To address extreme odds ratios and potential overfitting, a sensitivity analysis was conducted using Firth penalized logistic regression (**Supplementary Table 2**). This approach incorporates a Jeffreys prior penalty and provides more stable parameter estimates, particularly in the presence of small sample sizes or separation.

## Results

### Characteristics of the study population

**Figure 1:**
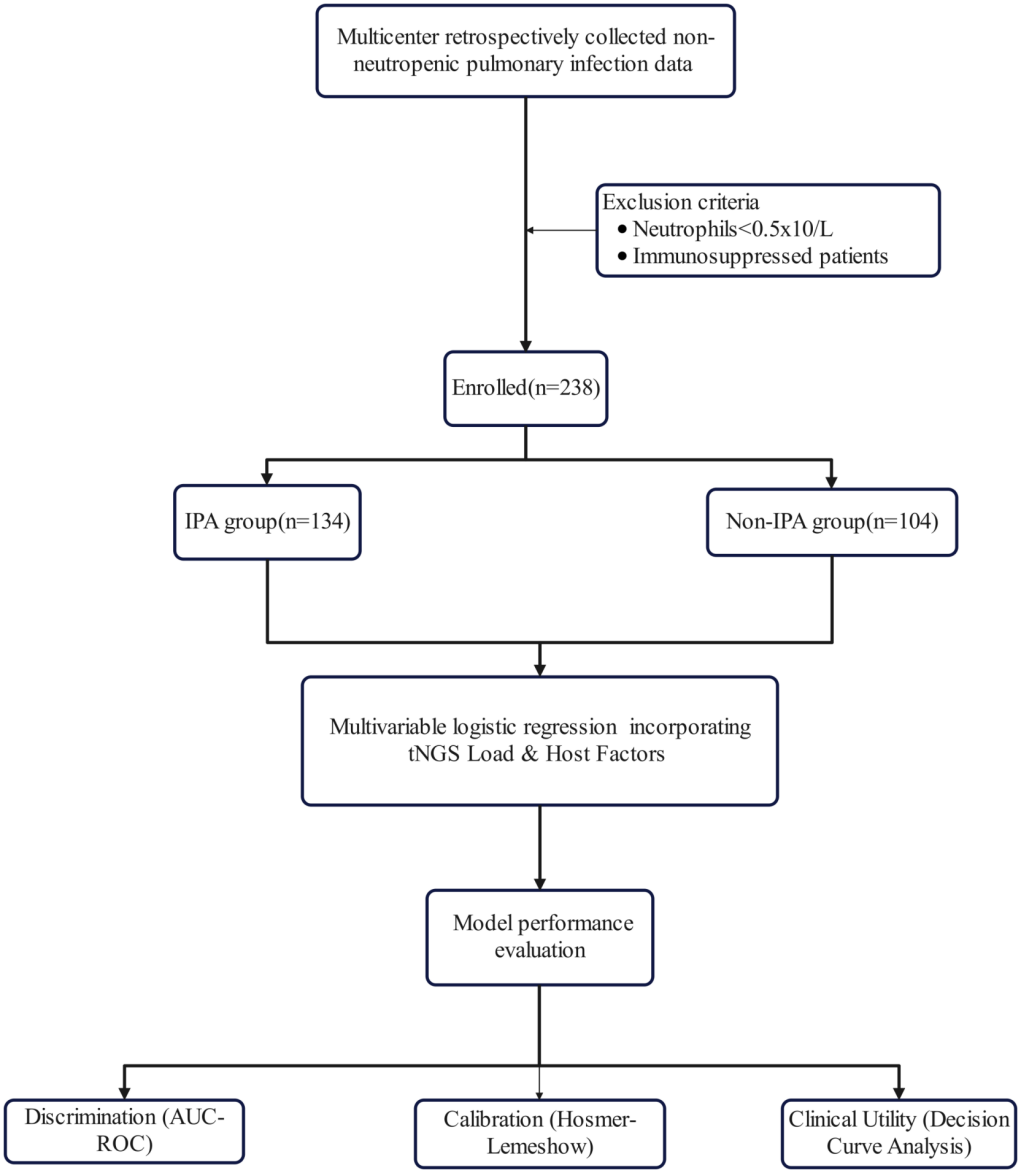
Study design and participant flow in the multicenter non-neutropenic cohort.

A total of 238 non-neutropenic adults with suspected pulmonary infection were enrolled across three tertiary care centers, including 134 patients with IPA and 104 with colonization (non-IPA) (**Figure 1**). Baseline characteristics are summarized in **Table 1**.

**Table 1:** Demographic, clinical, and laboratory characteristics of the study cohort.

Variable	IPA group (*n* = 134)	Non-IPA group (*n* = 104)	χ^2^/t	*P*
Age, years	66.35 ± 10.15	62.08 ± 13.71	2.763	0.006
Sex			0.119	0.730
Male	79 (59.0%)	59 (56.7%)		
Female	55 (41.0%)	45 (43.3%)		
PaO_2_/FiO_2_ ratio	212.00 (178.00,236.25)	307.50 (264.25,327.75)	-10.500	<0.001
ICU admission	97 (72.4%)	24 (23.1%)	56.968	<0.001
Smoking history	87 (64.9%)	44 (42.3%)	12.105	<0.001
Corticosteroid exposure	117 (87.3%)	36 (34.6%)	70.826	<0.001
Corticosteroid Exposure duration, days	7.00 (4.00,9.00)	0.00 (0.00,3.00)	-8.855	<0.001
Antimicrobial therapy				
Antibiotic treatment	131 (97.8%)	71 (68.3%)	39.671	<0.001
Antibiotic exposure duration, days	12.00 (8.75,21.00)	8.00 (0.00,12.25)	-6.151	<0.001
Voriconazole	120 (89.6%)	57 (42.5%)	37.084	<0.001
Other antifungal agents	60 (44.8%)	10 (9.6%)	34.868	<0.001
Laboratory examination				
White blood cells (×10^9^)	7.19 (5.61,8.97)	8.51 (6.64,11.38)	-2.758	0.006
Neutrophils (×10^9^)	4.86 (3.57,6.76)	5.79 (4.16,7.64)	-1.904	0.057
Lymphocytes (×10^9^)	2.16 (1.50,3.09)	2.49 (1.77,3.12)	-2.350	0.019
Procalcitonin (ng/mL)	0.27 (0.12,0.90)	0.09 (0.07,0.46)	-4.592	<0.001
Interleukin-6 (pg/mL)	14.71 (3.56,31.62)	8.03 (2.54,25.15)	-2.187	0.029
GM assay (BALF)	3.15 (2.32,4.30)	0.29 (0.23,0.42)	-13.226	<0.001
1,3-β-D-glucan assay (BALF)	64.00 (53.75,76.00)	33.00 (31.00,36.00)	-13.210	<0.001
BALF culture	73 (54.5%)	21 (20.2%)	28.803	<0.001
Symptoms				
Shortness of breath	98 (73.1%)	44 (42.3%)	23.121	<0.001
Chest pain	30 (22.4%)	8 (7.7%)	9.425	0.002
Chest tightness	103 (76.9%)	54 (51.9%)	16.226	<0.001
Hemoptysis	21 (15.7%)	2 (1.9%)	12.678	<0.001
Dyspnea	87 (64.9%)	34 (32.7%)	24.341	<0.001
Chest imaging				
Pleural effusion	87 (64.9%)	38 (36.5%)	18.922	<0.001
Nodular shadow	69 (51.5%)	39 (37.5%)	4.625	0.032
Patchy shadow	85 (63.4%)	38 (36.5%)	16.960	<0.001
Cavity	27 (20.1%)	4 (3.8%)	13.738	<0.001
Lung consolidation	107 (79.9%)	59 (56.7%)	14.834	<0.001
Pleural thickening	101 (75.4%)	48 (46.2%)	21.354	<0.001
Disease				
Viral pneumonia	76 (56.7%)	26 (25.0%)	24.052	<0.001
Bacterial infections	127 (94.8%)	38 (36.5%)	93.393	<0.001
Hypoproteinemia	98 (73.1%)	42 (40.4%)	25.928	<0.001
Diabetes	99 (73.9%)	23 (22.1%)	62.800	<0.001
Coronary heart disease	27 (20.1%)	28 (26.9%)	1.512	0.219
Respiratory disease	84 (62.7%)	52 (50.0%)	3.848	0.050
Outcome				
In-hospital mortality	46 (34.3%)	13 (12.5%)	14.964	<0.001
Clinical severity scores				
SOFA score	11.00 (10.00,14.00)	8.50 (8.00,11.00)	-7.356	<0.001
APACHE II score	18.00 (15.00,21.00)	13.00 (11.00,15.75)	-8.290	<0.001

Data are presented as mean ± SD, median (IQR), or n (%). *P* values were calculated using the Kruskal-Wallis test for continuous variables and the χ² test or Fisher’s exact test for categorical variables. *P* values are presented for descriptive, exploratory comparisons and have not been adjusted for multiple testing.

APACHE II, Acute Physiologic Assessment and Chronic Health Evaluation II; ICU, intensive care unit; PaO_2_/FiO_2_, arterial oxygen partial pressure/fractional inspired oxygen; SOFA, Sequential Organ Failure Assessment.

Patients with IPA were significantly older than non-IPA controls (mean difference, 4.3 years; 66.4 ± 10.2 *vs*. 62.1 ± 13.7 years; *P* = 0.006) and more frequently presented with critical illness. This was evidenced by substantially higher rates of intensive care unit (ICU) admission (absolute risk difference [ARD], 49.3%; risk ratio [RR], 3.1; 72.4% *vs*. 23.1%; *P* < 0.001), corticosteroid exposure (ARD, 52.7%; RR, 2.5; 87.3% *vs*. 34.6%; *P* < 0.001), and diabetes mellitus (ARD, 51.8%; RR, 3.3; 73.9% *vs*. 22.1%; *P* < 0.001).

Markers of respiratory dysfunction and systemic inflammation were also significantly more severe in the IPA group. Compared with non-IPA patients, those with IPA exhibited markedly lower PaO_2_/FiO_2_ ratios (median difference, −96; median, 212 *vs*. 308; *P* < 0.001), alongside higher circulating levels of procalcitonin (median difference, 0.18 ng/mL; 0.27 *vs*. 0.09 ng/mL; *P* < 0.001) and interleukin-6 (median difference, 6.7 pg/mL; 14.7 *vs*. 8.0 pg/mL; *P* = 0.029). Disease-severity scores were consistently elevated, including the Sequential Organ Failure Assessment (SOFA) score (median difference, 2.5 points; 11.0 *vs*. 8.5; *P* < 0.001) and the Acute Physiology and Chronic Health Evaluation II (APACHE II) score (median difference, 5.0 points; 18.0 *vs*. 13.0; *P* < 0.001).

Radiologically, IPA was associated with a significantly higher prevalence of cavitary lesions (RR, 5.3; 20.1% *vs*. 3.8%; *P* < 0.001), patchy opacities (RR, 1.7; 63.4% *vs*. 36.5%; *P* < 0.001), and pleural effusions (RR, 1.8; 64.9% *vs*. 36.5%; *P* < 0.001). Mycological indicators further distinguished the two groups: BALF galactomannan levels were markedly higher in the IPA group (median difference, 2.86; 3.15 *vs*. 0.29; *P* < 0.001), and Aspergillus-positive BALF cultures were more than 2.5-fold more frequent (RR, 2.7; 54.5% *vs*. 20.2%; *P* < 0.001). In-hospital mortality was also substantially increased among patients with IPA (RR, 2.7; 34.3% *vs*. 12.5%; *P* < 0.001).

### Development and diagnostic performance of the integrated model

A multivariable logistic regression model was constructed that incorporated normalized *Aspergillus* reads from BALF-tNGS together with host factors. Seven independent predictors of IPA were identified (**Table 2**): *Aspergillus* level (OR 0.21, 95% CI 0.09–0.51; *P* = 0.001), diabetes mellitus (OR, 25.64; 95% CI, 6.74–97.52; *P* < 0.001), corticosteroid exposure (OR, 24.15; 95% CI, 7.55–77.27; *P* < 0.001), bacterial co-infection (OR, 10.32; 95% CI, 2.51–42.39; *P* = 0.001), ICU admission (OR, 6.09; 95% CI, 2.05–18.04; *P=*0.001), nodular shadow on imaging (OR, 4.73; 95% CI, 1.65–13.58; *P* = 0.004), and positive BALF culture (OR, 14.59; 95% CI, 3.64–58.50; *P* < 0.001).

**Table 2:** Multivariate logistic regression analysis identifying independent predictors of invasive pulmonary aspergillosis.

Variable	B	SE	Wald	*P* value	Odds ratio (95% CI)
*Aspergillus* level	-1.558	0.452	11.877	0.001	0.211 (0.087, 0.511)
Diabetes mellitus	3.244	0.682	22.661	0	25.644 (6.743, 97.522)
Corticosteroid exposure	3.184	0.593	28.806	0	24.154 (7.55, 77.271)
Bacterial co-infection	2.334	0.721	10.494	0.001	10.324 (2.514, 42.388)
ICU admission	1.806	0.555	10.603	0.001	6.085 (2.052, 18.042)
Nodular shadow on imaging	1.553	0.538	8.32	0.004	4.726 (1.645, 13.578)
Positive BALF culture	2.68	0.709	14.301	0	14.586 (3.637, 58.504)

BALF, bronchoalveolar lavage fluid; ICU, intensive care unit.

**Figure 2:**
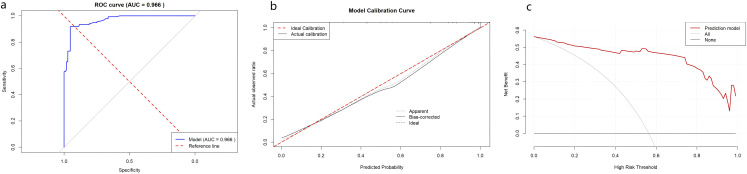
Diagnostic performance of the integrated model for invasive pulmonary aspergillosis. **(a)** Receiver operating characteristic curve demonstrating excellent discrimination (AUC = 0.966). **(b)** Calibration plot showing close agreement between predicted and observed probabilities (Hosmer–Lemeshow *P* = 0.472). **(c)** Decision-curve analysis indicating substantial clinical net benefit across a wide range of threshold probabilities.

The integrated model demonstrated excellent discrimination, with an AUC of 0.966 (**Figure 2a**). At the optimal cutoff (0.539), the model achieved a sensitivity of 91.8%, specificity 95.2%, PPV 96.1%, and NPV 90.0%. Calibration analysis confirmed close agreement between predicted and observed probabilities (Hosmer–Lemeshow χ²=7.61; *P* = 0.472; **Figure 2b**). Decision-curve analysis (DCA) showed consistent net clinical benefit across a broad range of threshold probabilities (**Figure 2c**).

**Figure 3:**
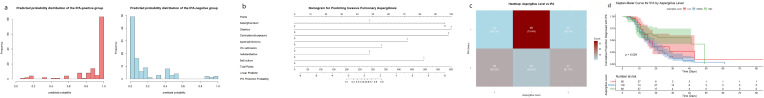
Visualization of the diagnostic model differentiating invasive pulmonary aspergillosis from colonization in non-neutropenic patients. **(a)** Predicted probability distribution in IPA-positive and IPA-negative groups. **(b)** Nomogram of the final multivariable prediction model integrating normalized *Aspergillus* reads with host factors. **(c)** Heatmap showing the distribution of IPA status across *Aspergillus*-load strata. **(d)** Kaplan–Meier curves illustrating time-to-IPA development by Aspergillus-load level (low-, medium-, and high-load groups).

The distribution of predicted probabilities and the corresponding nomogram for individualized risk estimation are presented in **Figures 3a, b**. The nomogram (**Figure 3b**) enables bedside application by assigning weighted points to each predictor, summing these to generate a total score (range, 0–600), and translating this score into an estimated probability of IPA. For example, application of the nomogram to a representative high-risk clinical profile (as detailed in the **Figure 3b** legend) results in a total score of approximately 230, corresponding to a predicted IPA probability exceeding 90%, thereby supporting early therapeutic decision-making.

### Integrated analysis of *Aspergillus* load and IPA risk

Patients were stratified by normalized *Aspergillus* read counts from BALF-tNGS into three groups: low (<177 RPM), medium (177–6,025 RPM), and high (>6,025 RPM) load groups (**Table 3**). The prevalence of IPA was highest in the medium-load group (75.4%, 89/118), significantly exceeding that in the low-load (36.7%) and high-load (38.3%) tiers (*P* < 0.001). Patients in the medium-load group also exhibited greater disease severity, with higher SOFA (11.0 [IQR, 9.0–13.0]) and APACHE II (17.0 [IQR, 14.0–19.0]) scores and lower PaO_2_/FiO_2_ ratios (median, 219), as well as more frequent corticosteroid exposure (78.0%), diabetes (63.6%), and bacterial co-infection (81.4%) than the other groups (all *P* < 0.001). Kaplan–Meier analysis revealed distinct IPA development trajectories across the three strata (log-rank *P* < 0.001; **Figure 3d**).

**Table 3:** Distribution of key model variables across *Aspergillus* load levels.

Variable	Overall (n=238)	Low load (n=60)	Medium load (n=118)	High load (n=60)	*P* value
Clinical characteristics, n (%)					
Diabetes mellitus	122 (51.3%)	18 (30.0%)	75 (63.6%)	29 (48.3%)	<0.001
Corticosteroid exposure	153 (64.3%)	30 (50.0%)	92 (78.0%)	31 (51.7%)	<0.001
Bacterial co-infection	165 (69.3%)	30 (50.0%)	96 (81.4%)	39 (65.0%)	<0.001
ICU admission	121 (50.8%)	20 (33.3%)	69 (58.5%)	32 (53.3%)	0.006
Nodular shadow on imaging	108 (45.4%)	20 (33.3%)	58 (49.2%)	30 (50.0%)	0.1
BALF culture result, n (%)					0.024
Negative	144 (60.5%)	44 (73.3%)	62 (52.5%)	38 (63.3%)	
Positive	94 (39.5%)	16 (26.7%)	56 (47.5%)	22 (36.7%)	
IPA diagnosis, n (%)	134 (56.3%)	22 (36.7%)	89 (75.4%)	23 (38.3%)	<0.001
Continuous variables, median (IQR)					
Corticosteroid exposure duration, days	4.0 (0.0, 8.0)	1.0 (0.0, 7.5)	6.0 (2.0, 9.0)	3.0 (0.0, 4.0)	<0.001
GM assay (BALF)	1.8 (0.3, 3.2)	0.4 (0.3, 2.2)	2.5 (1.1, 3.4)	0.4 (0.3, 3.3)	<0.001
1,3-β-D-glucan assay (BALF)	49.0 (34.0, 67.0)	36.5 (32.0, 50.0)	56.5 (46.0, 72.0)	36.0 (32.0, 64.5)	<0.001
Antibiotic exposure duration, days	10.0 (7.0, 18.0)	8.0 (1.0, 12.5)	12.0 (8.0, 19.0)	9.0 (4.0, 20.5)	<0.001
SOFA score	11.0 (8.0, 13.0)	9.0 (7.5, 13.0)	11.0 (9.0, 13.0)	11.0 (9.0, 13.0)	0.018
APACHE II score	16.0 (12.0, 19.0)	13.0 (11.0, 17.5)	17.0 (14.0, 19.0)	16.0 (14.0, 19.0)	<0.001
PaO_2_/FiO_2_ ratio	240.0 (204.0, 302.0)	280.0 (235.0, 320.0)	219.0 (188.0, 271.4)	253.0 (231.5, 312.0)	<0.001

*P* values were calculated using the χ² test for categorical variables and the Kruskal–Wallis rank-sum test for continuous variables. APACHE II, Acute Physiology and Chronic Health Evaluation II; BALF, bronchoalveolar lavage fluid; GM, galactomannan; ICU, intensive care unit; IPA, invasive pulmonary aspergillosis; SOFA, Sequential Organ Failure Assessment.

**Figure 4:**
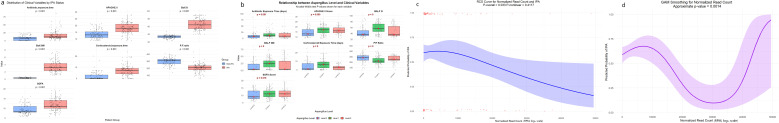
Distribution of clinical variables and association between normalized *Aspergillus* reads and invasive pulmonary aspergillosis risk. **(a)** Distribution of major clinical variables stratified by IPA status. **(b)** Relationship between *Aspergillus* load and key clinical variables. **(c)** Restricted cubic-spline analysis showing a significant overall association between normalized read count and IPA probability (*P-*overall=0.033). **(d)** Generalized additive-model smoothing curve demonstrating a consistent positive correlation (*P* = 0.0014).

Stratified analyses further revealed that host comorbidities substantially modified the relationship between fungal load and IPA risk (**Table 4**). Among patients with high *Aspergillus* load, diabetes increased IPA prevalence from 16.1% to 62.1%. In the medium-load group, concurrent bacterial co-infection increased IPA from 9.1% to 90.6%, whereas corticosteroid exposure and ICU admission raised rates from 46.2% to 83.7% and from 44.9% to 97.1%, respectively (**Figures 4a, b**).

**Table 4:** Stratified analysis of IPA prevalence by *Aspergillus* load level and clinical characteristics.

Variable		Low load			Medium load			High load		
		Total	IPA cases	Prevalence (%)	Total	IPA cases	Prevalence (%)	Total	IPA cases	Prevalence (%)
BALF culture	Negative	44	12	27.3%	62	33	53.2%	38	16	42.1%
	Positive	16	10	62.5%	56	56	100%	22	7	31.8%
Corticosteroid exposure	No	30	4	13.3%	26	12	46.2%	29	1	3.4%
	Yes	30	18	60%	92	77	83.7%	31	22	71%
Diabetes mellitus	No	42	7	16.7%	43	23	53.5%	31	5	16.1%
	Yes	18	15	83.3%	75	66	88%	29	18	62.1%
ICU admission	No	40	6	15%	49	22	44.9%	28	9	32.1%
	Yes	20	16	80%	69	67	97.1%	32	14	43.8%
Bacterial co-infection	No	30	5	16.7%	22	2	9.1%	21	0	0%
	Yes	30	17	56.7%	96	87	90.6%	39	23	59%
Nodular shadow on imaging	No	40	14	35%	60	42	70%	30	9	30%
	Yes	20	8	40%	58	47	81%	30	14	46.7%

Data are presented as counts and prevalence (%). BALF, bronchoalveolar lavage fluid; ICU, intensive care unit; IPA, invasive pulmonary aspergillosis.

When normalized *Aspergillus* reads were analyzed as a continuous variable, restricted cubic spline modeling demonstrated a significant overall association between read count and IPA probability (overall *P* = 0.033; *P* for nonlinearity=0.412), indicating an approximately linear dose–response trend (**Figure 4c**). A generalized additive model corroborated this finding, showing a consistent positive relationship (*P* = 0.0014) with widening uncertainty at extreme read values (**Figure 4d**). Collectively, these findings suggest that higher fungal read depth is associated with an increased IPA risk; however, its predictive strength is significantly modulated by host factors, including diabetes, corticosteroid exposure, and bacterial co-infection.

## Discussion

Growing evidence suggests that conditions commonly found in non-neutropenic hosts, such as COPD, diabetes mellitus, and hematologic malignancies, are independent risk factors for invasive fungal infections, as demonstrated in a seminal U.S. study (Menzin et al., 2009). In contrast to neutropenic patients, non-neutropenic individuals often exhibit non-specific or indolent manifestations, leading to under-recognition, diagnostic delay, and higher attributable mortality (Cornillet et al., 2006). In this context, the prompt and accurate identification of IPA is essential for optimizing antifungal therapy and improving outcomes (Bassetti and Bouza, 2017). In the present multicenter cohort, we integrated normalized *Aspergillus* reads from BALF-tNGS with host clinical factors to develop a diagnostic model capable of distinguishing IPA from colonization. Seven independent predictors were identified: *Aspergillus* load, diabetes, corticosteroid exposure, bacterial co-infection, ICU admission, presence of a nodular shadow on imaging, and positive BALF culture. The model demonstrated outstanding discrimination (AUC = 0.966), with sensitivity of 91.8%, specificity of 95.2%, PPV of 96.1%, and NPV of 90.0%. Calibration was excellent, and DCA confirmed substantial net clinical benefit across a wide range of threshold probabilities. Collectively, by coupling fungal molecular load with key host determinants, the integrated model substantially outperformed conventional microbiologic or radiologic criteria, establishing a framework for evidence-based interpretation of tNGS data in the diagnosis of IPA among non-neutropenic patients.

A key observation from this study was that the highest prevalence of IPA occurred in the medium–*Aspergillus*-load group (75.4%), significantly exceeding that observed in both the low-load (36.7%) and high-load (38.3%) strata. Patients in the medium-load group also exhibited greater disease severity, characterized by higher SOFA and APACHE II scores, as well as more frequent corticosteroid exposure (78.0%), diabetes mellitus (63.6%), and bacterial co-infection (81.4%) compared to those in the other groups. To further delineate the quantitative relationship between fungal molecular burden and IPA risk, both restricted cubic spline (RCS) and generalized additive model (GAM) analyses were performed, treating normalized *Aspergillus* reads as a continuous variable.

The RCS model revealed a significant overall association between read count and the probability of IPA (overall *P* = 0.033), whereas the test for nonlinearity was not significant (*P* for nonlinearity=0.412), indicating an approximately linear dose–response relationship. Consistently, the GAM smoothing curve showed a steadily increasing IPA probability with higher read counts (*P* = 0.0014), although confidence intervals widened at the extremes of the distribution. These findings suggest that, at the population level, higher fungal molecular signals generally correspond to an increased risk of IPA.

However, when the continuous variable was categorized into discrete strata (low, medium, and high) for multivariable modeling, a contrasting pattern emerged. After adjustment for host factors, including diabetes, corticosteroid exposure, bacterial co-infection, and ICU admission, a higher categorized fungal load was paradoxically associated with a lower odds ratio for IPA (OR = 0.21, 95% CI 0.09–0.51). This association remained robust in sensitivity analyses using Firth penalized regression, which reduced the magnitude of extreme odds ratios and mitigated the risk of overfitting. Rather than indicating a protective effect of higher fungal burden, this finding underscores the complex and context-dependent relationship between molecular fungal signals and invasive disease. Specifically, categorized fungal load captures only part of the pathogenic process and is strongly modulated by host susceptibility and clinical context. The divergent effects observed between continuous and categorized analyses reinforce the notion that tNGS-derived fungal read counts should not be interpreted in isolation but must be integrated with host factors to accurately distinguish invasive infection from colonization.

This seemingly inconsistent pattern highlights the complex, nonlinear relationship between fungal burden and invasive disease. Specifically, an elevated molecular load does not necessarily reflect tissue invasion (Fang et al., 2023); instead, it may represent an acute but predominantly noninvasive state, with residual fungal nucleic acids persisting after pathogen clearance (Żarczyńska et al., 2023). In contrast, moderate fungal loads may correspond to an active yet balanced state of invasion, reflecting a dynamic equilibrium between the host and pathogen. This interpretation is supported by the lower rate of IPA diagnosis in the high-load group, which may reflect limited viable fungal presence despite abundant nucleic acid signals (Sturny-Leclère et al., 2025). This phenomenon is analogous to that observed in tuberculosis, where cell-free mycobacterial DNA may persist long after viable bacilli have been eliminated (Hernández-Pando et al., 2000). Collectively, these results reinforce that fungal molecular abundance cannot be directly equated with bacterial burden and that the interpretation of tNGS-derived fungal load must be integrated with the host’s clinical context to distinguish true invasive infection from colonization. This principle forms the core rationale of the composite diagnostic model proposed in this study.

The present study further delineates the host determinants that modulate susceptibility to IPA in non-neutropenic patients. Diabetes mellitus, corticosteroid exposure, bacterial co-infection, and ICU admission emerged as the most influential risk factors in our multivariable model, each contributing distinct yet synergistic pathways of immune compromise. Diabetes was identified as a powerful predictor (OR = 25.6), consistent with the well-established association between hyperglycemia and impaired innate immune function. A sustained hyperglycemic milieu disrupts metabolic homeostasis, attenuating neutrophil chemotaxis, phagocytosis, and reactive oxygen species generation, thereby predisposing individuals to fungal invasion even in the absence of neutropenia (Thind et al., 2023). Corticosteroid therapy, another major determinant (OR = 24.2), exerts a dual influence by suppressing macrophage activity and enhancing fungal virulence through glucocorticoid-responsive gene expression in *Aspergillus* fumigatus (Peghin et al., 2014). Bacterial co-infection was also a frequent finding (69.3% overall) and amplified the risk of IPA nearly tenfold (OR = 10.3). Beyond diagnostic masking, bacterial infections and their empirical treatment with broad-spectrum antibiotics may perturb the airway microbiome and local immune tone, facilitating fungal proliferation and invasion. Similarly, ICU admission (OR = 6.1) represents a composite risk state encompassing critical illness, mechanical ventilation, and invasive procedures, compounded by environmental factors such as elevated humidity that promote *Aspergillus* spore persistence (Koulenti et al., 2023). Collectively, these findings highlight that non-neutropenic IPA arises not from a single dominant factor but from the cumulative disruption of multiple host defense barriers. Rigorous glycemic control, judicious corticosteroid use, and targeted fungal surveillance, particularly in ICU settings, are therefore imperative for early recognition and prevention in this expanding at-risk population.

tNGS offers several technical and clinical advantages that make it particularly suitable for diagnosing respiratory fungal infections. It employs multiplex PCR amplification of predefined pathogen targets, yielding high analytical sensitivity and specificity within 24 h (Li et al., 2022). A key methodological innovation of the present study is the use of normalized RPM to stratify fungal burden into cohort-based categories. This approach circumvents the interplatform variability inherent in absolute read counts and establishes a reproducible, semi-quantitative framework for comparing tNGS results across laboratories. By integrating this standardized fungal-load metric with host clinical variables, we developed a model that achieved an AUC of 0.966 and provided individualized risk estimates through a visual nomogram. The nomogram enables clinicians to translate multivariable probabilities into actionable decisions, facilitating early initiation or de-escalation of antifungal therapy based on patient-specific risk profiles. Collectively, these advances extend the clinical interpretability of tNGS data, bridging the gap between molecular signal quantification and bedside decision-making. A nomogram score exceeding the validated threshold should prompt strong consideration of antifungal therapy, whereas a lower score may support treatment de-escalation in the appropriate clinical context, in conjunction with ongoing clinical reassessment.

Despite its robust multicenter design and rigorous analytical validation, this study has several limitations. First, the retrospective design may have introduced inherent selection and information biases, and the timing of BALF collection and antifungal exposure was not standardized across centers. Prior antifungal therapy may have confounded the observed relationship between fungal load and IPA risk, underscoring the need for prospective studies with standardized pre-sampling treatment windows. Second, although internal bootstrapping confirmed model robustness, external validation using prospectively enrolled, geographically diverse cohorts and alternative sequencing platforms is necessary to establish generalizability. Third, the diagnostic classification of IPA was based on consensus definitions rather than histopathologic confirmation in all cases, which may have led to the misclassification of borderline presentations. Fourth, the quantitative interpretation of tNGS reads remains partly assay-dependent; despite normalization by RPM, residual interplatform variability and batch effects cannot be entirely excluded. The absence of data on fungal viability and precise timing of antifungal therapy relative to BALF sampling further limits interpretation; prior antifungal exposure may reduce detectable fungal DNA, resulting in load misclassification and attenuation of its association with IPA risk. In addition, the percentile-based fungal-load categorization, while pragmatic, requires validation in external cohorts. Fifth, several predictors incorporated into the model, most notably ICU admission, corticosteroid exposure, and bacterial co-infection, are clinically correlated. Although stepwise selection and bootstrap resampling were applied to enhance model stability, and formal collinearity diagnostics demonstrated no evidence of problematic multicollinearity (all variance inflation factors < 3.5), residual clinical interdependence among predictors cannot be fully excluded. Accordingly, the large odds ratios observed for diabetes mellitus and corticosteroid exposure should be interpreted with caution, as they may partly reflect cohort-specific risk profiles and may not be directly transferable to other populations. Although the Hosmer–Lemeshow test suggested good overall calibration, this test has limited sensitivity to detect overfitting. Indeed, bootstrap-derived calibration slopes indicated moderate optimism in the original model, which improved after application of Firth penalized regression, highlighting the importance of cautious interpretation of absolute risk estimates. Finally, because this study was conducted in tertiary hospitals in China, the findings may not be directly generalizable to other healthcare systems or patient populations with differing pathogen spectra and antifungal exposure patterns. Future prospective multicenter investigations incorporating serial sampling, quantitative fungal viability assessments, and cross-platform standardization are warranted to refine and externally validate the proposed diagnostic model.

## Conclusions

This multicenter study established and internally validated an integrated diagnostic model that combines BALF-tNGS data with key host clinical factors to distinguish IPA from airway colonization in non-neutropenic patients. The model demonstrated excellent discrimination and clinical utility, outperforming conventional microbiologic and radiologic criteria. Normalized Aspergillus reads derived from tNGS correlated with IPA risk but required contextual interpretation alongside host determinants such as diabetes, corticosteroid exposure, and bacterial co-infection. By providing a standardized, semi-quantitative framework for interpreting Aspergillus molecular signals, this approach bridges the gap between molecular diagnostics and bedside decision-making. Prospective multicenter validation is warranted to confirm its applicability across diverse populations and sequencing platforms.

## Data Availability

The original contributions presented in the study are included in the article/**Supplementary Material**. Further inquiries can be directed to the corresponding authors.
