# Supplementary material for: Integrative diagnosis of invasive pulmonary aspergillosis in non-neutropenic patients using BALF-tNGS–derived Aspergillus load and host risk factors: a multicenter study
Source: Front Cell Infect Microbiol. 2026 Feb 12;16:1739837. doi: 10.3389/fcimb.2026.1739837 (PMC12935974; doi:10.3389/fcimb.2026.1739837)
Supplement: Supplementary file 1 [file Table1.docx]

| Patient group | Total (n) | Host factors present | Clinical/radiologic criteria met | Mycologic evidence | Essential criteria met |
| --- | --- | --- | --- | --- | --- |
| Proven IPA | 12 | Not required for proven cases | Not required for proven cases | Yes | Histopathologic evidence or culture from a sterile site |
| Probable IPA | 122 | Yes | Yes | Yes | All of the following: (1) host factors; (2) compatible clinical and radiologic criteria; (3) mycological evidence (positive culture or galactomannan) |
| Non-IPA (colonization or alternative diagnosis) | 104 | Varied | Varied (see breakdown below) | Varied (see breakdown below) | Did not meet the criteria for proven or probable invasive pulmonary aspergillosis |

Supplementary Table 1. Detailed classification of all 238 enrolled patients according to the 2024 FUNDICU consensus criteria for invasive pulmonary aspergillosis.
