# Supplementary material for: Integrative diagnosis of invasive pulmonary aspergillosis in non-neutropenic patients using BALF-tNGS–derived Aspergillus load and host risk factors: a multicenter study
Source: Front Cell Infect Microbiol. 2026 Feb 12;16:1739837. doi: 10.3389/fcimb.2026.1739837 (PMC12935974; doi:10.3389/fcimb.2026.1739837)
Supplement: Supplementary file 2 [file Table2.docx]

Supplementary Table 2. Comparison of odds ratios derived from conventional logistic regression and Firth penalized regression.

| **Variable** | **Original model OR (95% CI)** | **Firth penalized regression OR (95% CI)** | **Change (%)** |
| --- | --- | --- | --- |
| Aspergillus level (medium vs. low) | 0.21 (0.09–0.51) | 1.49 (0.39–5.89) | +609.5 |
| Aspergillus level (high vs. low) | 0.21 (0.09–0.51) | 0.21 (0.09–0.51) | 0.0 |
| Diabetes mellitus | 25.64 (6.74–97.52) | 15.50 (5.20–46.20) | −39.5 |
| Corticosteroid exposure | 24.15 (7.55–77.27) | 12.30 (4.80–31.50) | −49.1 |
| Bacterial co-infection | 10.32 (2.51–42.39) | 8.50 (3.20–22.60) | −17.6 |
| ICU admission | 6.08 (2.05–18.04) | 5.20 (2.10–12.90) | −14.5 |
| Nodular shadow on imaging | 4.73 (1.65–13.58) | 4.10 (1.90–8.90) | −13.3 |
| Positive BALF culture | 14.59 (3.64–58.50) | 9.80 (3.50–27.40) | −32.8 |

Notes:

Odds ratios (ORs) and 95% confidence intervals (CIs) were estimated using the original multivariable logistic regression model and a sensitivity analysis based on Firth penalized regression. Percentage change indicates the relative difference in OR magnitude between the two modeling approaches. Firth regression applies a penalized likelihood to reduce small-sample bias and instability associated with extreme estimates, generally yielding more conservative effect sizes. In the original model, *Aspergillus* level was specified as an ordinal variable, and the reported OR represents the change in odds of invasive pulmonary aspergillosis per one-level increase. In contrast, the Firth model treated *Aspergillus* level as a categorical variable, estimating medium- and high-level effects separately relative to the low-level reference category.
